# Older adults and individuals with Parkinson’s disease control posture along suborthogonal directions that deviate from the traditional anteroposterior and mediolateral directions

**DOI:** 10.1038/s41598-024-54583-y

**Published:** 2024-02-19

**Authors:** Madhur Mangalam, Damian G. Kelty-Stephen, Ivan Seleznov, Anton Popov, Aaron D. Likens, Ken Kiyono, Nick Stergiou

**Affiliations:** 1 Division of Biomechanics and Research Development, Department of Biomechanics, and Center for Research in Human Movement Variability, University of Nebraska at Omaha, Omaha, NE 68182 USA; 2 Department of Psychology, State University of New York at New Paltz, New Paltz, NY 12561 USA; 3 Graduate School of Engineering Science, Osaka University, Osaka, 560-8531 Japan; 4 Department of Electronic Engineering, Igor Sikorsky Kyiv Polytechnic Institute, Kyiv, 03056 Ukraine; 5 Faculty of Applied Sciences, Ukrainian Catholic University, Lviv, 79011 Ukraine; 6 Graduate School of Engineering Science, Osaka University, Osaka, 560-8531 Japan; 7 Department of Department of Physical Education, and Sport Science, Aristotle University, 570 01 Thessaloniki, Greece

**Keywords:** Diagnostic markers, Diagnostic markers, Parkinson's disease

## Abstract

A rich and complex temporal structure of variability in postural sway characterizes healthy and adaptable postural control. However, neurodegenerative disorders such as Parkinson’s disease, which often manifest as tremors, rigidity, and bradykinesia, disrupt this healthy variability. This study examined postural sway in young and older adults, including individuals with Parkinson’s disease, under different upright standing conditions to investigate the potential connection between the temporal structure of variability in postural sway and Parkinsonism. A novel and innovative method called oriented fractal scaling component analysis was employed. This method involves decomposing the two-dimensional center of pressure (CoP) planar trajectories to pinpoint the directions associated with minimal and maximal temporal correlations in postural sway. As a result, it facilitates a comprehensive assessment of the directional characteristics within the temporal structure of sway variability. The results demonstrated that healthy young adults control posture along two orthogonal directions closely aligned with the traditional anatomical anteroposterior (AP) and mediolateral (ML) axes. In contrast, older adults and individuals with Parkinson’s disease controlled posture along suborthogonal directions that significantly deviate from the AP and ML axes. These findings suggest that the altered temporal structure of sway variability is evident in individuals with Parkinson’s disease and underlies postural deficits, surpassing what can be explained solely by the natural aging process.

## Introduction

Parkinson’s disease is a progressive neurodegenerative condition of the dopaminergic pathways that manifest as resting tremors, rigidity, and bradykinesia^1–4^. Individuals with Parkinson’s disease also commonly experience issues with posture, specifically with orientation (i.e., maintaining proper alignment with gravity) and stabilization or maintaining a balance against external forces^5–8^. The difficulties in orientating relative to gravity may be caused by rigidity, which often starts in the limbs but can also affect the trunk and neck. The problems with stabilization, primarily due to tremors typically emerging later in the disease progression and bradykinesia, can result in postural instability even during simple upright standing^9^. Although individuals with Parkinson’s disease tend to lean forward while standing upright, they are highly susceptible to falling backward even under minimally destabilizing conditions—a phenomenon referred to as “retropulsion”^10,11^. Retropulsion occurs partly due to axial rigidity and poor trunk coordination, which impair patients’ stability when faced with backward force. Individuals with Parkinson’s disease demonstrate a narrower margin of stability when sustaining an upright standing position compared to age-matched healthy adults; this measurement stands out as a widely accepted and objective gauge of dynamic stability^12^. This diminished stability is evident irrespective of whether the person assumes a narrow or wide stance^13^. This postural instability also hampers their ability to transition between different postures^14,15^. Due to this postural instability, individuals with Parkinson’s disease face an increased risk of falls and injuries, significantly impeding their mobility, independence, and quality of life^11,16,17^.

Mounting evidence underscores the intricate nature of Parkinson’s disease pathophysiology, surpassing the confines of the dopaminergic system. Non-dopaminergic systems, specifically neurotransmitter pathways like cholinergic^18–20^, noradrenergic^21,22^, and serotoninergic^23–25^, are increasingly acknowledged for their pivotal role in disrupting postural control mechanisms. Viewing Parkinson’s disease through the lens of accelerated aging^26,27^ introduces a nuanced perspective. It is essential to note, however, that although aging is typically linked to neuronal loss, particularly in regions like the substantia nigra^28,29^, the mechanisms and expression patterns of neuronal decline in Parkinson’s disease (primarily characterized by synucleinopathy^30–32^) diverge significantly from those observed in normal aging. Nevertheless, the impact of aging on these neuronal pathways may still play a role in exacerbating postural deficits among individuals grappling with Parkinson’s disease. Consequently, recognizing these non-dopaminergic contributions adds layers of intricacy to our comprehension of the pathophysiological landscape of postural control impairment in Parkinson’s disease, setting the stage for exhaustive investigations and targeted therapeutic interventions.

In the present study, we employ the theoretical footing of the optimal movement variability hypothesis, which has proposed that a healthy and adaptable postural control system is characterized by a complex but organized temporal structure in postural sway variability^33–35^. However, as individuals age or experience neurological diseases, their postural systems become compromised. In such cases, postural sway may exhibit either consistent variations over time, leading to stiff and highly predictable behavior, or dissimilar and random variations, resulting in erratic and unfocused behavior^36–38^. Conversely, a healthy and highly adaptable postural system displays optimal postural sway variability. Growing evidence suggests that individuals with Parkinson’s disease exhibit a highly predictable temporal structure of postural sway compared to healthy controls, indicating reduced flexibility^39–41^. Notably, it is well-established that individuals with Parkinson’s disease cannot respond to destabilizing forces along the AP axis; their stance width influences this capacity^13,42^. Furthermore, individuals who experience freezing of gait demonstrate adequate postural control and sensory integration abilities when maintaining a stable posture. However, they exhibit impaired postural control along the AP axis when they voluntarily shift their weight^43^. Conversely, during sit-to-stand transitions and obstacle crossing, they display increased postural instability along the ML axis^44,45^. While previous research has primarily focused on quantifying the magnitude of postural sway and assessing it individually along the AP and ML axes, these findings suggest that the direction in which reduced flexibility in stabilizing posture manifests in individuals with Parkinson’s disease is task-specific. Therefore, a deeper exploration of postural sway that examines its temporal structure of variability along the actual directions involved in postural control can provide a better understanding of the sources of these postural deficits^46–50^.

Traditionally, analyzing human postural sway involves studying the 2D planar trajectory of the postural center of pressure (CoP), which typically exhibits characteristics of fractional Brownian motion (fBm)^51,52^. In this context, fBm is a statistical model that characterizes the CoP trajectory, with its sample-to-sample fluctuations adhering to fractional Gaussian noise (fGn). The strength of these temporal correlations in CoP fluctuations is quantified using a fractal scaling exponent called the Hurst exponent, *fGn*^53–55^. A substantial body of empirical evidence solidly establishes the significant role of the variability of fractal scaling in diverse postural adaptations^46–50^. This perspective gains theoretical validation as well. It is acknowledged that the intermittent controller skillfully choreographs unique sway patterns via a cyclical process^56^. This process emerges from the intricate dance between deliberate, slow movements tracing the stable manifold of an upright equilibrium resembling a saddle and the spiraling motions that take us away from this central point. Remarkably, by artfully blending these two unstable regimes, a remarkable degree of dynamic stability can be attained^56^. Crucially, this mode of postural control hinges on the asymmetric distribution of fractal scaling, quantifying the strength of these temporal correlations between these two directions. For instance, postural sway exhibits larger fractal scaling along the AP axis and smaller scaling exponent along the ML axis^52,57,58^, implying more active (feedback based) and passive (feedforward based) control strategies along the AP and ML axes, respectively. Thus, postural control appears to strategically allocate its temporal variability across the AP and ML axes, mirroring its approach to simpler, time-insensitive variability^59^.

In this traditional model of analyzing human postural sway, the CoP planar trajectory is projected onto orthogonal directions along the AP and ML axes to facilitate this analysis. This projection assumes that postural control primarily occurs along these two anatomical axes, drawing inspiration from the inverted pendulum model. According to this model, the body rotates around one or more “hinges” aligned in the AP axis^60–62^. This conception of postural control faces a formidable challenge, as sway proves to be more flexible and fluid than our initial expectations suggested^63^. While the AP and ML axes are vital measurements derived from the force plate, relying solely on stability parameters along the AP and ML axes may erroneously create the impression that those orientations universally apply to every individual^64,65^. To maintain postural balance, continuous and comprehensive integration of motor control in multiple directions is essential^66–68^. While previous studies have employed multi-directional protocols to investigate postural control in healthy adults^58,69–73^ and individuals with Parkinson’s disease^13,74^, the interpretation of these data has predominantly adhered to the anatomical AP and ML axes. For example, the sway directional index, employed to quantify postural sway’s directional characteristics, calculates the path length ratio along either the AP or ML axis to the total path length^69,75^. An experimental approach delves into postural sway along the AP and ML axes in response to surface translations occurring at oblique angles to these axes^13^. This conventional approach arises from practical considerations driven by the output provided by force platforms, adherence to anatomical conventions, and the simplification inherent in inverted pendulum models. However, this prevalent methodological choice warrants critical consideration as it may oversimplify the intricate nature of postural control dynamics, potentially limiting our comprehensive understanding of the underlying mechanisms involved.

We propose that the spatial distribution of sway variability could serve as a valuable lens to discern disparities in postural control, particularly when distinguishing individuals with Parkinson’s disease from older adults. We can break free from strict rules like AP vs. ML axis and right angles to better understand postural control. Instead, we can look at the angles and directions between the smallest and largest fractal scaling patterns. This helps us tell the difference between strong and not-so-strong postural control. This innovative approach employs oriented fractal scaling component analysis (OFSCA)—a novel analytical technique—that decomposes two-dimensional trajectories into the directions corresponding to the most active and passive control^76^. This individualized portrayal of postural control was then applied to a comprehensive analysis of two publicly available postural sway datasets featuring three distinct groups: healthy young adults, older adults, and individuals with Parkinson’s disease, both with and without Levodopa medication^77,78^. The participants’ balance was assessed across four conditions of upright standing, including motionless standing for 30 s on a stable surface with both eyes open and closed and on an unstable surface under similar vision conditions. Our primary objective was to investigate the impact of Parkinson’s disease on the directional characteristics of posture sway. We hypothesized that individuals with Parkinson’s disease would exhibit altered directional postural sway characteristics compared to healthy young adults. Considering Parkinson’s disease as a form of accelerated aging^26,27^, we also anticipated similar but less severe changes in the directional characteristics of postural sway in older adults. Building on the principles of optimal movement variability, our secondary hypothesis focused on the critical role of an optimal spatiotemporal structure of postural sway in maintaining postural stability. Consequently, we expected that changes in the temporal structure of variability in postural sway would predict alterations in the directional characteristics of postural sway within those populations.

## Results

The OFSCA method^76^ was employed to investigate the autocorrelation properties of 2D CoP planar trajectories in 32 Parkinson’s patients ($M\pm SD$ age: $66\pm 10$ years; 8 women) in both off-medication and on-medication conditions, 22 healthy older adults ($67\pm 8$ years; 11 women), and 27 healthy younger adults ($28\pm 5$ years; 12 women) in four different postural conditions: standing on a rigid surface with eyes open, standing on a rigid surface with eyes closed, standing on an unstable surface with eyes open, and standing on an unstable surface with eyes closed.

### An overview of the oriented fractal scaling component analysis (OFSCA)

**Figure 1:**
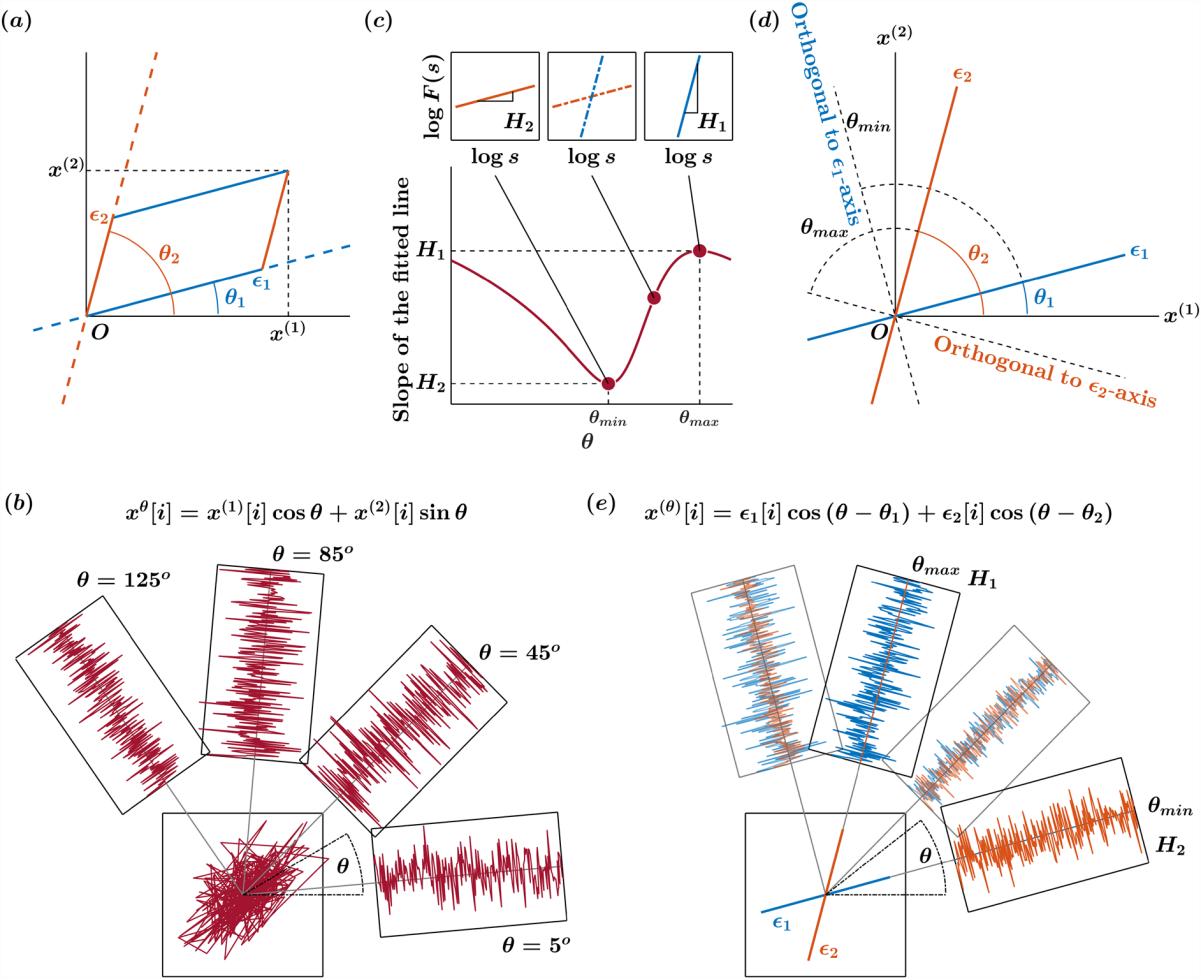
Principle illustration of the orientation detection of angle-dependent temporally correlated components $\bigl \{\epsilon _{1}[i]\bigl \}$ and $\bigl \{\epsilon _{2}[i]\bigl \}$ in the $\bigl (x^{(1)},x^{(2)}\bigl )$ plane. The premise of the OFSCA is that the observed 2D planar CoP trajectory displays spatially distributed temporal correlations. The directions with the largest temporal correlations define the axes of influence for posture control, exemplified by trajectories $\epsilon _{1}$ and $\epsilon _{2}$ at angles $\theta _{1}$ and $\theta _{2}$ relative to the horizontal reference direction (**a**). To unveil the inherent patterns within the initial trajectories $\epsilon _{1}$ and $\epsilon _{2}$ derived from the observed 2D planar trajectory—represented in this instance as fractional Gaussian noise, fGn—the OFSCA procedure commences with a transformation of the observed 2D trajectory. (**b**). This transformation expands the trajectory to encompass a comprehensive set spanning all angles within $0\le \theta <\pi$. Subsequently, the DDMA analysis is employed to gauge the strength of temporal correlations in these extended trajectories across each angle (**c**). The directions associated with the maximum and minimum strengths of temporal correlations $H_{1}$ and $H_{2}$ are obtained within this framework. Identifying the original components entails pinpointing the directions corresponding to these scaling exponents’ maximum and minimum values, designated as $\theta _{max}$ and $\theta _{min}$, respectively. Notably, these values consistently run orthogonal to the original orientations of the components (**d**). Ultimately, the orientations of $H_{1}$ and $H_{2}$ are used to reconstruct the actual 2D planar trajectory comprising $\epsilon _{1}$ and $\epsilon _{2}$ (**e**).

The OFSCA method assesses the angle-dependent scaling properties within trajectories through a sophisticated technique known as higher-order detrended moving average (DMA) analysis, specifically DDMA. While we offer an in-depth exposition of the OFSCA method within our “Methods” section, complete with an illustrative simulation employing a 2D fractional Brownian motion trajectory, allow us to provide a concise overview of this analysis. Within the domain of 2D planar postural CoP trajectories, our prevailing model is built upon two pivotal assumptions. Initially, we assert that a 2D CoP planar trajectory can be exhaustively characterized by two distinct sample paths of fractional Brownian motion (fBm). By the same token, fluctuations in a 2D CoP planar trajectory can be exhaustively characterized by two distinct sample paths of fractional Gaussian noise (fGn). Subsequently, we posit that these two components exist in perpetual orthogonality, implying that the scaling characteristics of each angular component remain steadfast and immune to rotational transformations. It is noteworthy, however, that this presumption of isotropy may be a more infrequent occurrence rather than a ubiquitous phenomenon, as the natural, planar postural sway trajectories may not consistently demonstrate isotropic behavior. In essence, this signifies that the fluctuations exhibit a spatial distribution of temporal correlations, and the directions showcasing the most robust temporal correlations delineate the axes along which posture control exerts its influence. This is vividly illustrated by the trajectories $\epsilon _{1}$ and $\epsilon _{2}$ at angles $\theta _{1}$ and $\theta _{2}$ respectively, relative to the horizontal reference direction in Fig. 1a.

To unveil the intrinsic patterns within the original trajectories $\epsilon _{1}$ and $\epsilon _{2}$ from the observed 2D planar trajectory (in this case, represented by the fGn), the OFSCA procedure initiates by a transformation of the observed 2D trajectory. It extends this trajectory into a comprehensive set encompassing all angles within the range $0\le \theta <\pi$, vividly depicted in Fig. 1b. Following this transformation, the DDMA analysis comes into play, measuring the strength of temporal correlations embedded within these expanded trajectories along each angle. Within this context, the directions associated with the maximal and minimal strengths of temporal correlations, conveniently labeled as $H_{1}$ and $H_{2}$ within Fig. 1c, are then isolated. Detecting the original components requires identifying the directions corresponding to these scaling exponents’ maximum and minimum values, denoted as $\theta _{max}$ and $\theta _{min}$. Remarkably, these values are consistently orthogonal to the original orientations of the components, as demonstrated in Fig. 1d. Ultimately, the orientations of $H_{1}$ and $H_{2}$ are used to reconstruct the actual 2D planar trajectory comprising of $\epsilon _{1}$ and $\epsilon _{2}$ and the corresponding directions, as depicted in Fig. 1e.

### Directional variability in postural control across age and Parkinson’s disease

**Figure 2:**
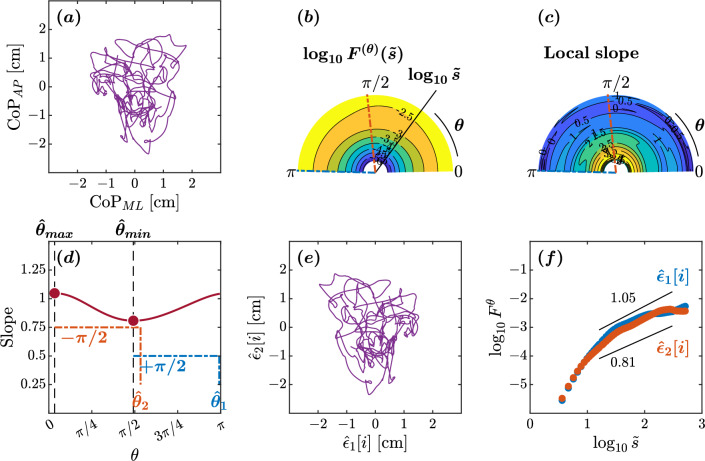
Orientation decomposition of the CoP trajectory of a representative younger adult maintaining an upright balance on an unstable surface with eyes closed. (**a**) CoP along the anatomical AP and ML axes. (**b**) $\theta$-dependent heterogeneity in CoP fluctuations, indicated by the angle dependence of $\log _{10}^{(\theta )}F^{(\theta )}(\tilde{s})$ vs. $\log _{10}\tilde{s}$, where $\tilde{s}\sim s/1.93$ in the second order DDMA. (**c**) $\theta$-dependence of the local slopes of $\log _{10}^{(\theta )}F^{(\theta )}(\tilde{s})$ vs. $\log _{10}\tilde{s}$, indicating the spatial distribution of temporal correlations. (**d**) $\theta$-dependence of the slope in the range of $1.2<\log _{10}{\tilde{s}}<2.5$. (**e**) Reconstructed CoP along the original directions of postural control, $\hat{\epsilon }_{1}[i],\hat{\epsilon }_{2}[i]$. (**f**) Fluctuation functions of CoP along the original directions of postural control, $\hat{\epsilon }_{1}$ with $\hat{\theta }_{1}=178^{\circ }$ and $\hat{\epsilon }_{2}$ with $\hat{\theta }_{2}=96^{\circ }$.

**Figure 3:**
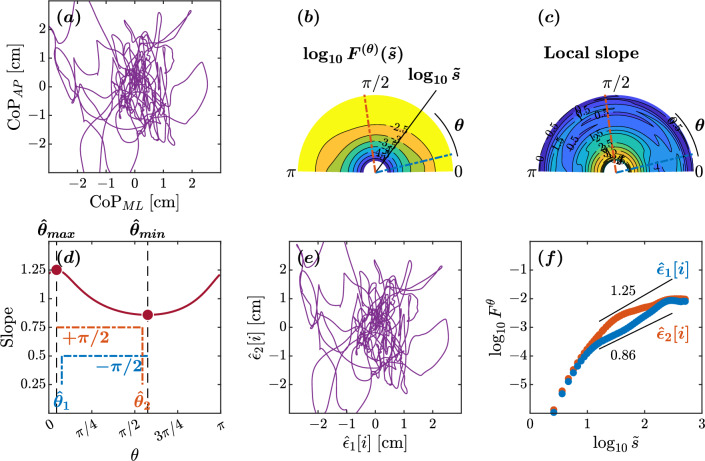
Orientation decomposition of the CoP trajectory of a representative healthy older adult maintaining an upright balance on an unstable surface with closed eyes. (**a**) CoP along the anatomical AP and ML axes. (**b**) $\theta$-dependent heterogeneity in CoP fluctuations, indicated by the angle dependence of $\log _{10}^{(\theta )}F^{(\theta )}(\tilde{s})$ vs. $\log _{10}\tilde{s}$, where $\tilde{s}\sim s/1.93$ in the second order DDMA. (**c**) $\theta$-dependence of the local slopes of $\log _{10}^{(\theta )}F^{(\theta )}(\tilde{s})$ vs. $\log _{10}\tilde{s}$, indicating the spatial distribution of temporal correlations. (**d**) $\theta$-dependence of the slope in the range of $1.2<\log _{10}{\tilde{s}}<2.5$. (**e**) Reconstructed CoP along the original directions of postural control, $\hat{\epsilon }_{1}[i],\hat{\epsilon }_{2}[i]$. (**f**) Fluctuation functions of CoP along the original directions of postural control, $\hat{\epsilon }_{1}$ with $\hat{\theta }_{1}=14^{\circ }$ and $\hat{\epsilon }_{2}$ with $\hat{\theta }_{2}=98^{\circ }$.

**Figure 4:**
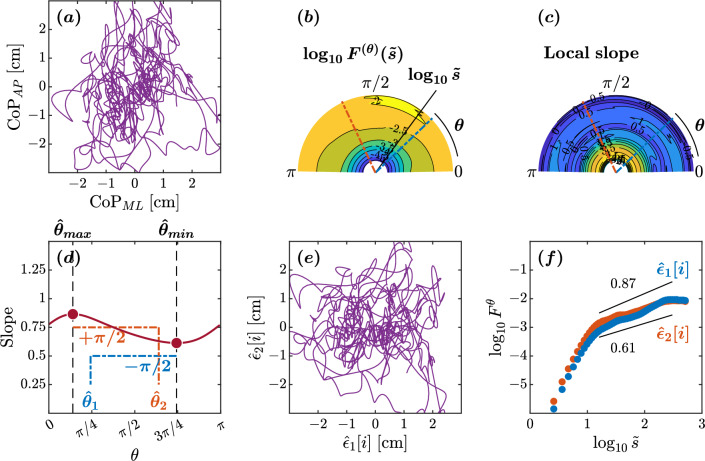
Orientation decomposition of the CoP trajectory of a representative individual with Parkinson’s disease off medication maintaining an upright balance on an unstable surface with eyes closed. (**a**) CoP along the anatomical AP and ML axes. (**b**) $\theta$-dependent heterogeneity in CoP fluctuations, indicated by the angle dependence of $\log _{10}^{(\theta )}F^{(\theta )}(\tilde{s})$ vs. $\log _{10}\tilde{s}$, where $\tilde{s}\sim s/1.93$ in the second order DDMA. (**c**) $\theta$-dependence of the local slopes of $\log _{10}^{(\theta )}F^{(\theta )}(\tilde{s})$ vs. $\log _{10}\tilde{s}$, indicating the spatial distribution of temporal correlations. (**d**) $\theta$-dependence of the slope in the range of $1.2<\log _{10}{\tilde{s}}<2.5$. (**e**) Reconstructed CoP along the original directions of postural control, $\hat{\epsilon }_{1}[i],\hat{\epsilon }_{2}[i]$. (**f**) Fluctuation functions of CoP along the original directions of postural control, $\hat{\epsilon }_{1}$ with $\hat{\theta }_{1}=44^{\circ }$ and $\hat{\epsilon }_{2}$ with $\hat{\theta }_{2}=115^{\circ }$.

**Figure 5:**
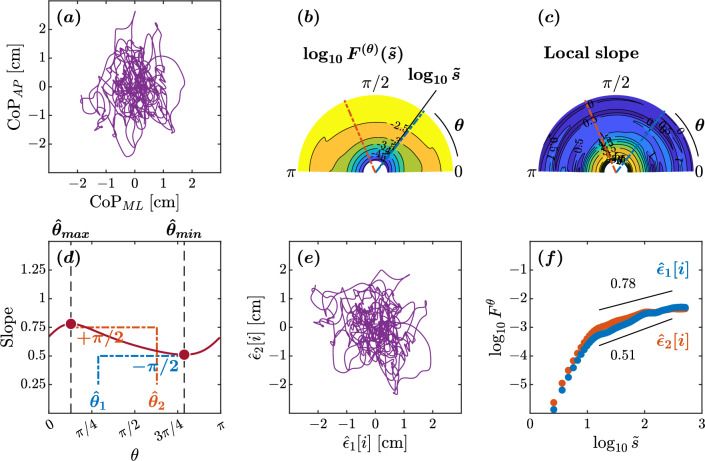
Orientation decomposition of the CoP trajectory of a representative individual with Parkinson’s disease on medication maintaining an upright balance on an unstable surface with eyes closed. (**a**) CoP along the anatomical AP and ML axes. (**b**) $\theta$-dependent heterogeneity in CoP fluctuations, indicated by the angle dependence of $\log _{10}^{(\theta )}F^{(\theta )}(\tilde{s})$ vs. $\log _{10}\tilde{s}$, where $\tilde{s}\sim s/1.93$ in the second order DDMA. (**c**) $\theta$-dependence of the local slopes of $\log _{10}^{(\theta )}F^{(\theta )}(\tilde{s})$ vs. $\log _{10}\tilde{s}$, indicating the spatial distribution of temporal correlations. (**d**) $\theta$-dependence of the slope in the range of $1.2<\log _{10}{\tilde{s}}<2.5$. (**e**) Reconstructed CoP along the original directions of postural control, $\hat{\epsilon }_{1}[i],\hat{\epsilon }_{2}[i]$. (**f**) Fluctuation functions of CoP along the original directions of postural control, $\hat{\epsilon }_{1}$ with $\hat{\theta }_{1}=52^{\circ }$ and $\hat{\epsilon }_{2}$ with $\hat{\theta }_{2}=113^{\circ }$.

The plots depicted in Figs. 2, 3, 4, and 5 represent the orientation decomposition of the CoP trajectory of a healthy young adult, older adult, individuals with Parkinson’s disease off medication, and individuals with Parkinson’s disease on medication, maintaining an upright balance on an unstable surface with eyes closed. This particular postural condition was deliberately chosen as it presented the most challenging task, expected to result in the most pronounced anisotropy in postural sway. In other words, the selected posture was expected to lead to the most noticeable directional differences in the individual’s swaying pattern. We evaluated the angle dependence of $F^{(\theta )}(\tilde{s})$ for the original CoP trajectory (Figs. 2a, 3a, 4a, 5a) over the range of $0\le \theta <\pi$ in increments of $\pi /179$ rad, specifically indicating the spatial distribution of temporal correlations (Figs. 2b,c, 3b,c, 4b,c, 5b,c). We set the scaling range $1.2<\log _{10}{\tilde{s}}<2.5$ (from 0.16 to 3.2 s) and estimated the slopes of linear regressions (Figs. 2d, 3d, 4d, 5d) to find two representative orientations.

In the healthy adult maintaining balance on an unstable surface with eyes closed, the minimum and maximum slopes were observed at $\hat{\theta }_{min}=88^{\circ }$ and $\hat{\theta }_{max}=9^{\circ }$, respectively (Fig. 2d). This yielded estimated orientations of $\hat{\theta }_{1}=178^{\circ }$ and $\hat{\theta }_{2}=96^{\circ }$. The scaling behaviors of the reconstructed original components $\hat{\epsilon }_{1}[i]$ and $\hat{\epsilon }_{2}[i]$ exhibited a nontrivial difference (Fig. 2d–f). A crossover point around $\log _{10}{\tilde{s}}=1.2$ was observed in both components $\hat{\epsilon }{1}$ and $\hat{\epsilon }{2}$ (Fig. 2f). In the scaling range $1.2\le \tilde{s}<2.5$, the scaling exponent $H=1.05$ for the orientation $\hat{\epsilon }_{1}$ suggests high levels of complexity and predictability in postural sway. The extracted CoP component possesses the 1/*f* pink noise characteristics, reflecting the complexity and hierarchical organization of the underlying control mechanisms. Likewise, the $H=0.81$ for the orientation $\hat{\epsilon }_{2}$ indicates less predictable postural sway. Finally, the $\hat{\epsilon }_{2}$ orientation was close to the ML axis, and the $\hat{\epsilon }_{1}$ orientation was close to the AP axis, suggestive of healthy planar control of posture.

In the older adult maintaining balance on an unstable surface with eyes closed, the minimum and maximum slopes were observed at $\hat{\theta }_{min}=108^{\circ }$ and $\hat{\theta }_{max}=8^{\circ }$, respectively (Fig. 3d). This yielded estimated orientations of $\hat{\theta }_{1}=14^{\circ }$ and $\hat{\theta }_{2}=98^{\circ }$. The scaling behaviors of the reconstructed original components $\hat{\epsilon }_{1}[i]$ and $\hat{\epsilon }_{2}[i]$ exhibited a noteworthy difference (Fig. 3d–f). In the scaling range $1.2\le \tilde{s}<2.5$, the scaling exponent $H=1.25$ for the orientation $\hat{\epsilon }_{1}$ suggests intact control along the major direction of control. In contrast, the scaling exponent $H=0.86$ for the orientation $\hat{\epsilon }_{2}$ indicates a loss of temporal correlations along the minor direction of control. The $\hat{\epsilon }_{1}$ and $\hat{\epsilon }_{2}$ orientations deviated significantly from the AP and ML axes, with a suborthogonal orientation relative to each other, suggestive of altered postural control in this older adult. In short, this older adult showed anisotropy in postural control compared to the healthy young adult discussed above.

In the individual with Parkinson’s disease off medication maintaining balance on an unstable surface with eyes closed, the minimum and maximum slopes were observed at $\hat{\theta }_{min}=134^{\circ }$ and $\hat{\theta }_{max}=25^{\circ }$, respectively (Fig. 4d). This yielded estimated orientations of $\hat{\theta }_{1}=44^{\circ }$ and $\hat{\theta }_{2}=115^{\circ }$. In the scaling range $1.2\le \tilde{s}<2.5$, the scaling behaviors of the reconstructed original components $\hat{\epsilon }_{1}[i]$ and $\hat{\epsilon }_{2}[i]$ exhibited a nontrivial difference (Fig. 4e,f). The estimated minimum and maximum slopes of 0.61 and 0.87 indicate a considerable loss of temporal correlations due to the loss of coordinated control activity along both directions. The $\hat{\epsilon }_{1}$ orientation was suborthogonal to the $\hat{\epsilon }_{2}$ orientation and deviated considerably from the ML axis. In short, this individual with Parkinson’s disease also showed anisotropy in postural control and a greater loss of temporal correlations in postural sway.

In the same individual with Parkinson’s disease on medication, the minimum and maximum slopes were observed at $\hat{\theta }_{min}=142^{\circ }$ and $\hat{\theta }_{max}=23^{\circ }$, respectively (Fig. 5d). This yielded estimated orientations of $\hat{\theta }_{1}=52^{\circ }$ and $\hat{\theta }_{2}=113^{\circ }$. The scaling behaviors of the reconstructed original components $\hat{\epsilon }_{1}[i]$ and $\hat{\epsilon }_{2}[i]$ exhibit a nontrivial difference (Fig. 5e,f). The estimated minimum and maximum slopes of 0.51 and 0.78 indicate a considerable loss of temporal correlations along both directions. Again, the $\hat{\epsilon }_{1}$ orientation was suborthogonal to the $\hat{\epsilon }_{2}$ orientation and deviated considerably from the ML axis. In short, this individual with Parkinson’s disease also showed anisotropy in postural control and a greater loss of temporal correlations in postural sway. The Levodopa treatment achieved no gains in postural control in this individual with Parkinson’s disease.

### Older adults and individuals with Parkinson’s control posture along suborthogonal directions that deviate from the traditional anteroposterior and mediolateral directions

**Figure 6:**
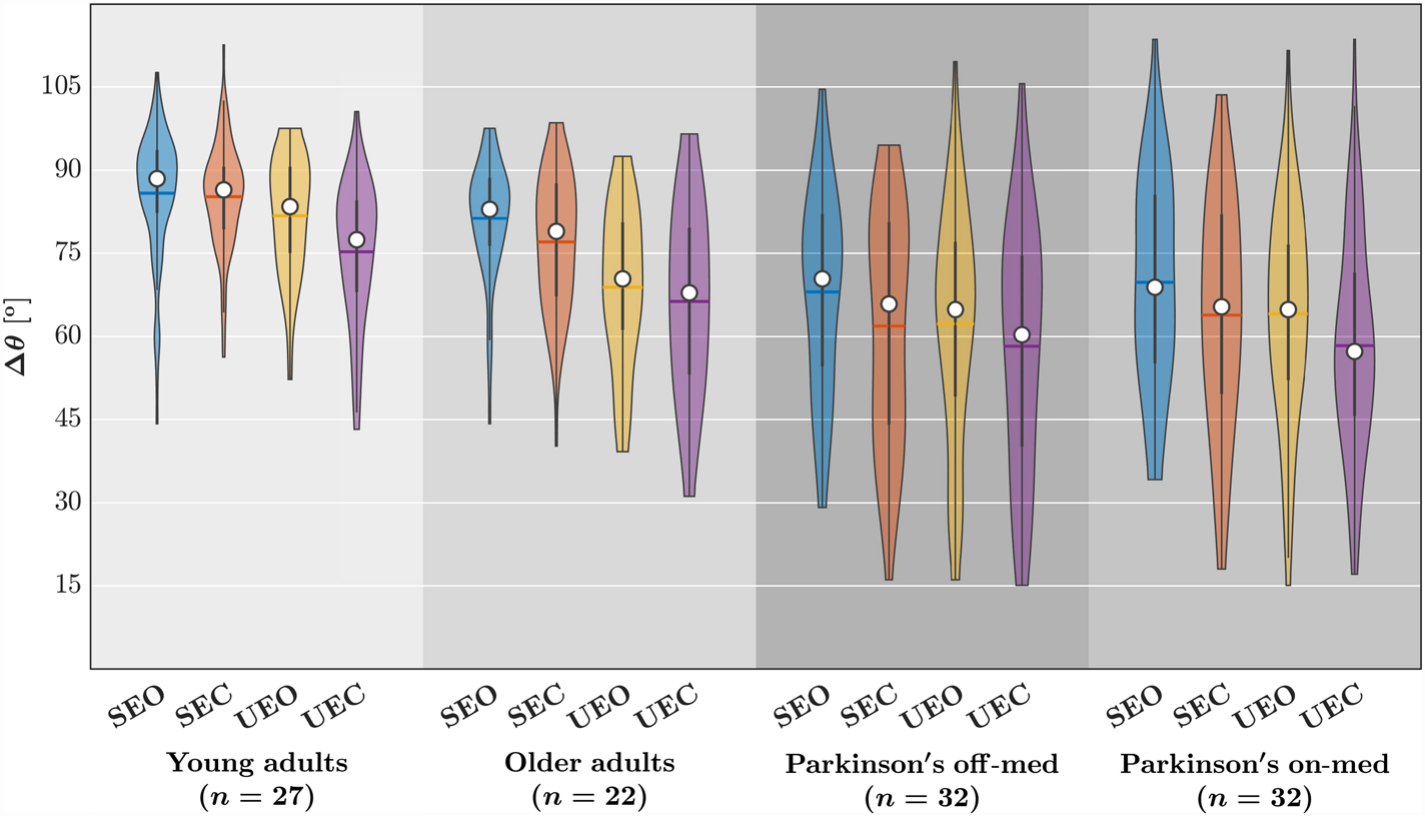
The relative orientation of the major and minor axes of postural control, $\Delta \theta =\hat{\theta }_{1}\sim \hat{\theta }_{2}$, for each group and postural condition. Horizontal bars indicate group *mean*, and white circles indicate group *median*. *SEC* stable, eyes closed, *UEO* unstable, eyes open, *UEC* unstable, eyes closed.

The linear mixed-effect model (described in Statistical Analysis) returned coefficients for each covariate indicating the average change in $\Delta \theta$ associated with group membership in class variable values or a unit increase in the *B* corresponding continuous variable. Each coefficient had a corresponding standard error *SE* indicating the variation around the associated average change in $\Delta \theta$; we report the estimated coefficients from the linear mixed-effect model in what follows in the form “$B\pm SE$” and noting, in parentheses, the corresponding *t*-statistic, which is equal to *B*/*SE* as well as the *p*-value estimate according to Satterthwaite’s method. At the group level (Table 1, Fig. 6), the eyes closed condition reduced $\Delta \theta$ by $3.762\pm 1.650^{\circ }$ ($t=-2.280,p=0.023$), and the unstable postural condition reduced $\Delta \theta$ by $6.449\pm 1.698^{\circ }$ ($t=-3.779,p=1.512\times 10^{-4}$). Compared to healthy young adults, older adults showed a reduction in $\Delta \theta$ by $6.308\pm 3.025^{\circ }$ ($t=-2.085,p=0.038$), and individuals with Parkinson’s disease showed thrice the magnitude of reduction in $\Delta \theta$ than did older adults, that is, by $20.776\pm 2.756^{\circ }$ ($t=-7.537,p=1.180\times 10^{-12}$). This threefold excess of $\Delta \theta$ in the Parkinson’s disease group above and beyond the healthy older adult group indicates that individuals with Parkinson’s disease exhibit altered suborthogonal axes of postural control beyond that predictable from healthy aging. To stress this point, we note that the increase in $\Delta \theta$ with Parkinson’s is almost five standard errors greater than for the healthy older adults. Levodopa medication did not help restore healthy postural control in individuals with Parkinson’s disease who went to show comparable distortion of postural control compared to healthy young adults: $18.652\pm 2.773^{\circ }$ ($t=-6.727,p=1.390\times 10^{-10}$). Interestingly, the observed divergence in outcomes based on diagnosis might have been substantial enough to overshadow the impact of task sensitivity. Notably, closing eyes did not yield any discernible effects for any participants, and individuals with Parkinson’s disease exhibited no response to experimental destabilization, exemplified by standing on foam blocks. However, it is noteworthy that older adults without a diagnosis displayed a significant reduction in $\Delta \theta$ of $-5.092\pm 2.465^{\circ }$ ($t=-2.066,p=0.040$), surpassing the magnitude observed in younger adults but still aligning with the same directional trend. This moderate reduction in older adults, in contrast to the absence of such an effect in individuals with Parkinson’s disease, may suggest a strategic adaptation in postural control. Specifically, the substantial decrease in $\Delta \theta$ under stable conditions could be deemed maladaptive for maintaining stability. Paradoxically, this pronounced reduction in baseline values might create a narrower scope for adaptive, smaller decreases in $\Delta \theta$ among healthy older adults.

**Table 1:** Outcomes of the LME examining the influence of group and postural conditions on $\Delta \theta$.

Factor	B $\pm SE$	*t*	*P*
(Intercept)	$88.292 \pm 3.762$	23.472	$<2.000 \times 10^{-16}$
GroupOA	$-6.308 \pm 3.025$	$-2.085$	0.038
GroupPN	$-20.776 \pm 2.756$	$-7.537$	$1.180 \times 10^{-12}$
GroupPM	$-18.652 \pm 2.773$	$-6.727$	$1.390 \times 10^{-10}$
EyesClosed	$-3.762 \pm 1.650$	$-2.280$	0.023
Unstable	$-6.449 \pm 1.698$	$-3.799$	$1.520 \times 10^{-4}$
$H_{1}$	$-13.601 \pm 3.403$	$-3.997$	$6.780 \times 10^{-5}$
$H_{2}$	$10.579 \pm 3.916$	2.702	0.007
GroupOA:EyesClosed	$0.154 \pm 2.462$	0.063	0.950
GroupPN:EyesClosed	$-1.332 \pm 2.240$	$-0.595$	0.552
GroupPM:Eyesclosed	$-2.137 \pm 2.243$	$-0.953$	0.341
GroupOA:Unstable	$-5.092 \pm 2.465$	$-2.066$	0.040
GroupPN:Unstable	$2.219 \pm 2.240$	0.991	0.322
GroupPM:Unstable	$1.015 \pm 2.243$	0.452	0.651

Fitted model: $$\Delta \theta \sim \textrm{Group}\times (\textrm{EyesClosed}+\textrm{Unstable})+H_{1}+H_{2}+(1|\textrm{Participant})$$.

*OA* older adults, *PN/PM* individuals with Parkinson’s disease, off Levodopa/on Levodopa.

Notably, across all participant groups—healthy young and older adults and individuals with Parkinson’s disease on and off medication, the reduction in $\Delta \theta$ depended on the strength of temporal correlations along the two directions ($H_{1}$ and $H_{2}$, respectively). An increase in $H_{2}$ was associated with an increase in $\Delta \theta$ ($t=2.702,p=0.007$). In contrast, an increase in $H_{1}$ was associated with a reduction in $\Delta \theta$ ($t=-3.997,p=6.780\times 10^{-5}$). Importantly, we found no interaction effects of $H_{2}$ and $H_{1}$ with aging and Parkinsonism, further confirming that the change in the temporal structure of postural variability moderated the observed changes in $\Delta \theta$. Regardless of aging or Parkinsonism, the inherent spatial variability in temporal correlations within postural control suggests potential implications for the spatial arrangement of suborthogonal axes in the postural control system. In summary, the loss of temporal structure in postural variability shaped the supposedly suborthogonal directional characteristics of postural control in older adults and individuals with Parkinson’s disease.

## Discussion

This study aimed to understand the directional characteristics of postural instability in individuals with Parkinson’s disease. We questioned the assumption that measuring postural control in the AP and ML axes provides sufficient information on the directional aspects of the subject’s stability. Presenting these values can create the impression that the directions are relevant to the subject’s instability, even though they provide no insight into the specific direction of instability. To address this issue, we studied the directional characteristics of the postural CoP using the OFSCA method^76^. The OFSCA technique allowed us to dissect the two distinct components of 2D CoP planar trajectories distinguished by their varying temporal correlations while pinpointing the characteristic directions associated with each. The OFSCA first projected a 2D CoP planar trajectory across various angles, subsequently estimating the scaling exponents inherent in the resulting time series and detecting the original components involved, identifying that these scaling exponents’ maximum and minimum values are invariably orthogonal to the components’ original orientations. We found that healthy young adults control posture along two orthogonal directions, closely aligned with the traditional anatomical AP and ML axes. In contrast, older adults and individuals with Parkinson’s disease control posture along suborthogonal directions that significantly deviate from the AP and ML axes. Moreover, changes in the temporal structure of variability in postural sway predict changes in the directional characteristics of the temporal structure of variability. These results indicate a close relationship between Parkinsonism and changed temporal structure of variability in postural sway, which may explain postural abnormalities beyond what can be accounted for by age alone.

### Relaxing commitment to orthogonal AP- and ML-directionality reveals novel information about postural control in individuals with Parkinson’s disease

The present work uncovers Parkinson’s disease-related differences in postural control that emerge when we move away from strict adherence to orthogonal AP- and ML-directionality. Previous studies on postural asymmetry in Parkinson’s disease have primarily examined the theoretical significance of AP and ML axes, often assuming orthogonality implicitly^79–81^. For instance, one hypothesis posits that increased ML activity observed in individuals with Parkinson’s disease reflects their effort to compensate for impaired movements along the AP axis, maintaining stability during quiet standing^81^. Alternatively, it has been suggested that individuals with Parkinson’s disease experience more pronounced impairments in lateral postural stability due to the basal ganglia’s pivotal role in trunk coordination. While lateral stability predominantly depends on hip and trunk control, AP stability primarily involves ankle control^79,80^. Our present findings introduce nuances to this understanding of postural deficits in Parkinson’s disease, unveiling a distinct alteration in the directional characteristics of postural control. Specifically, the spatial distribution of fractal scaling exponents indicates that individuals with Parkinson’s disease display suborthogonal postural control, deviating from the orthogonal postural control observed in healthy young adults. This highlights distinctive postural control dynamics in Parkinson’s disease and suggests amplification of age-related changes in control mechanisms, supporting the idea of Parkinson’s disease being akin to accelerated aging^82–84^. Notably, no significant effects were observed when closing the eyes or standing on an unstable surface in relation to these directions, implying that these directional characteristics are more influenced by an individual’s inherent model of postural control rather than specific task constraints, at least regarding these two manipulations.

While most postural instability associated with Parkinson’s disease is evident during dynamic activities such as walking, turning, or rising from a chair^85–87^, our current investigation uniquely focuses on analyzing directional distortion in postural control exclusively during quiet standing. The ability to maintain postural control in a standing position is foundational to the skill of walking. Without a stable standing posture, the fundamental prerequisite for effective walking is compromised, highlighting the integral relationship between static postural control and the subsequent dynamic act of walking. That being said, acknowledging the delimited scope of this research, which concentrates solely on static postural conditions, is imperative. Furthermore, the employed OFSCA method is not presently tailored to address the intricacies of directional control within more dynamic tasks. Despite these inherent limitations, our primary objective is to refine and broaden this methodology to encompass a more comprehensive array of dynamic tasks. Anticipating its enhancement, we envisage the OFSCA method’s increased applicability for the meticulous examination of postural control in dynamic settings, not only among individuals with Parkinson’s disease but also within the broader spectrum of movement disorders affecting postural control.

The study’s most intriguing and significant contribution lies in examining temporal correlations as moderators of suborthogonal postural control in older adults and individuals with Parkinson’s disease. Parkinson’s disease is characterized by three primary symptoms: resting tremors, rigidity, and bradykinesia (slow movement)^1,3,4^, all of which are linked to variability. Surprisingly, despite some exceptions, research has paid limited attention to the temporal structure of this variability and its potential implications for specific deficits. The optimal movement variability hypothesis, which posits that a healthy and adaptable system should exhibit a predictable and intricate temporal structure in postural sway^33–35^, offers valuable insights. It is reasonable to speculate that the weakening of these temporal correlations contributes to the suboptimal postural control observed in individuals with Parkinson’s disease. Indeed, our findings strongly suggest that the mechanisms for detecting and controlling postural instability may diverge between individuals with Parkinson’s disease and their healthy counterparts. This divergence becomes evident when examining the direction displaying the most pronounced temporal correlations, which implies a diminished feedback mechanism for returning to the equilibrium point of posture. Notably, individuals with Parkinson’s disease exhibit weaker temporal correlations in this direction than normal controls, aligning with a more rigid control mechanism prevailing in Parkinson’s disease. In what follows, we discuss the possible physiological basis of this rigidity.

### Exploring the vestibular basis of suborthogonal postural control in aging and Parkinson’s disease

The findings immediately raise a fundamental question: what underlies the sensorimotor deficits responsible for suborthogonal postural control in older adults and individuals with Parkinson’s disease? We contend that this suborthogonality may signify compensatory behavior or serve as a coping strategy for vestibulopathy—a condition commonly associated with natural aging and Parkinson’s disease, and our assertion gains support from the finding that closing eyes did not affect the directional control of posture. Early investigations into vestibular function in Parkinson’s disease consistently revealed deficits in the vestibular contributions to postural control. These studies encompassed examinations of the vestibular-ocular reflexes (VORs), which play a crucial role in maintaining stability during actions involving continuous head and eye movements^88–91^. Neurophysiological research delving into posture and cervical vestibular-evoked myogenic potentials (cVEMPs), a measure of electromagnetic potentials generated by neck muscles in response to sound stimulation, further corroborated these findings^92–99^. Moreover, individuals with Parkinson’s disease also show abnormal visual perception of the vertical in the upright position and less accurate perception of forward tilt when standing on a motion platform^100–103^, further corroborating the presence of compromised vestibular function in individuals with Parkinson’s disease. Furthermore, additional multimodal studies further support those findings and indicate that individuals with Parkinson’s disease may encounter deficits in the integration of sensory information in the central nervous system, including vestibular functioning^104–110^.

Neurophysiological findings strongly suggest that the neuropathological impact of Parkinson’s disease extends into the central vestibular system^111^. This extension is likely to compromise certain vestibular reflexes and influence autonomic, limbic system, and cortical pathways responsible for conveying vestibular information^112–117^. Substantial evidence supports the presence of Parkinsonian neuropathological alterations within the vestibular nucleus complex. Those alterations include the presence of Lewy bodies^116^, reduced levels of nonphosphorylated neurofilament, and an increase in lipofuscin^117^. Also, there are indications of reduced cholinergic input to the thalamus^114^, which is particularly intriguing in light of the observed decline in connectivity with the pedunculopontine tegmental nucleus in Parkinson’s disease^112^. Notably, the pedunculopontine tegmental nucleus plays a pivotal role as a primary source of cholinergic input. This nucleus houses vestibular-responsive neurons^118^ and undergoes significant changes in the number of acetylcholine-containing neurons following bilateral vestibular loss^119^.

Postural instability stemming from compromised vestibular function in individuals diagnosed with Parkinson’s disease is a significant concern. This impairment results in distorted postural control, potentially influencing their directional stability and amplifying their vulnerability to falls^10,11^. Notably, age-related degeneration of vestibular function is a well-documented phenomenon in the context of healthy aging^120,121^. Additionally, older adults who experience multiple non-syncopal falls often exhibit peripheral vestibular dysfunction, emphasizing the relevance of this sensory system in maintaining balance and preventing falls among the elderly population^122^. Fractal properties in sensorimotor behavior serve as critical indicators of both impaired functional capacity and the internal mechanisms that enable individuals to sustain performance despite external constraints^123–125^. When vestibular function is compromised, it can lead to a reduction in postural adaptability, characterized not only by a decrease in fractal scaling but also by diminished suborthogonal control of posture in various directions^123–125^. This multifaceted impact underscores the intricate relationship between vestibular function, postural control, and stability maintenance, with important implications for Parkinson’s disease and the aging population.

### Postural deficits in aging and Parkinson’s disease: a unified understanding

Aging stands as the foremost peril in the realm of Parkinson’s disease^82,126^, elevating the odds of its manifestation in an individual^127^ and ensuring that those who grapple with Parkinson’s disease in their later years, face intensified motor impairment, levodopa responsiveness, gait and postural deficits, and the looming specter of dementia^128^. Remarkably, a tapestry of similarities weaves Parkinson’s disease and the natural aging process together (reviewed by Rodriguez et al.^26^). The discernible pattern of selective brain cell loss^82^ and accumulation of $\alpha$-synuclein protein^129^ characterizes aging and Parkinson’s disease alike. Furthermore, the very transformations that define normal aging are implicated in the genesis of Parkinson’s disease, including heightened protein aggregation^130^, augmented oxidative stress^131^, dwindling mitochondrial function^132^, proteasome dysfunction^133^, and impaired autophagy^134^. Considering the parallels between the normal aging process and Parkinson’s disease, a compelling argument emerges, proposing that Parkinson’s disease could be an inherent byproduct of the aging process itself^26^. Our work reinforces this notion. We have uncovered that older adults and those grappling with Parkinson’s disease exhibit posture control along non-traditional, suborthogonal axes, distinct from the conventional anteroposterior and mediolateral directions. Significantly, individuals with Parkinson’s disease showcased a similar deficit, albeit to a more pronounced degree. The Parkinson’s disease group displays a threefold surplus of $\Delta \theta$ compared to the healthy older adult group, revealing distinct alterations in suborthogonal axes of postural control beyond what can be attributed to normal aging. Emphasizing this distinction, it is noteworthy that the rise in $\Delta \theta$ among individuals with Parkinson’s is nearly five times the standard errors observed in the healthy older adult group. One salient parameter intimately linked to falls in the elderly population is the heightened variability in step width^135^. Furthermore, this age-associated alteration in step width variability is notably contingent upon the direction of motion. Notably, the anteroposterior dimension exhibits a significantly greater degree of variability than the mediolateral dimension, a phenomenon also discernible within the context of ambulation^136,137^. In light of our current findings, in conjunction with these observations, it becomes evident that a common underlying factor may contribute to both postural instability and falls during walking in older adults and individuals afflicted with Parkinson’s disease. This shared observation not only underscores the potential for interventions aimed at retarding the aging process as a protective measure for neural health and postural control in the management of Parkinson’s disease but also hints at the possibility that rehabilitation strategies designed to ameliorate posture in older adults and individuals with Parkinson’s disease may have overlapping foundations.

### Moving beyond temporal variability: spatial (multi)fractality in postural control

This work points to future directions in elaborating multifractal characterization of postural control from a temporal domain into a spatial domain. Crucially, most multifractal treatments of postural control rely on recognizing that fractal scaling of postural control is not constant but fluctuates across time. The fBm-type designation of postural sway reflects only an approximate description of the general tendency to show temporal correlations beyond additive white Gaussian noise (awGn)^138^. The body of empirical literature examining fractal scaling in postural control has, on the contrary, revealed that fractal scaling could vary across time, yielding “multiple” fractal-scaling patterns, that is, a diversity of fractal-scaling patterns so systematic as to warrant the generalization to so-called “multifractal” formalisms. The variation of fractal scaling across time has long been an important indicator of health estimable from postural fluctuations^123,139,140^. Nevertheless, now we see that it is not just the variation across time of fractal scaling but also the variation of this temporal correlation across space that could unlock new insights from measurable postural sway. Indeed, the initial attempts to examine postural fluctuations solely along one or another orthogonal direction were just a first step—each of which is simply one dimension at a time and never the entirety of the full-bodied postural system moving its center of pressure. With the development of the OFSCA tool, we can generalize the multifractal framework to the two-dimensional plane of CoP fluctuations. Indeed, previous research has already investigated fractal scaling as an expression of the two-dimensional dispersion through a best-fitting ellipse of CoP positions (e.g.,^141–143^). However, these prior methods have been, in effect, statements about a spatial histogram of CoP positions frozen over time. The limitation of such histogram-based methods is that CoP reflects a concerted flow of postural control mechanisms that shift and migrate through the two dimensions from one equilibrium point to another^48^. Multifractal generalization of those spatial-histogram methods can, at minimum, estimate different fractal dimensions for different-sized events, and such multifractal histogram treatments can reveal necessary signatures of time-evolving movement coordination across two-dimensional support surfaces^144^. The present work suggests there is yet further to learn from imbuing these spatial methods with information about temporal correlation and its directionality. The multifractal modeling of human movement variability may soon catch up with the multidimensional scope of multifractal modeling in other empirical domains^145^.

### Directional dependency of CoP trajectory in Parkinson’s disease

In summary, comprehending postural control in Parkinson’s disease demands a nuanced, multidimensional approach considering individualized patient deficits and treatment responses. The therapeutic impact of levodopa on posture can vary significantly among individuals, with some experiencing remarkable improvements while others witness more modest or limited benefits. As the efficacy of levodopa diminishes over time due to disease progression and motor complications, leading to a decreasing therapeutic response, continuous monitoring and adaptive treatment strategies become imperative. Our investigation revealed notable limitations in levodopa’s effects on planar posture control, a recognized notion in scientific literature^146–149^. These findings underscore the need for a personalized investigative approach that leverages temporal correlations and other factors to unravel the complexities of postural coordination and address levodopa therapy’s limitations. Ongoing research and exploration of novel interventions are paramount for optimizing the management of posture-related symptoms in Parkinson’s disease, a goal our study actively contributes to. Postural instability is a pivotal feature of Parkinson’s disease, often culminating in falls, injuries, and substantial morbidity. Unfortunately, our understanding of the root causes of this postural dyscontrol remains incomplete. While previous studies in individuals with Parkinson’s disease primarily focused on measuring motor responses to external perturbations^13,146,150–152^, they often present challenges and yield conflicting results due to individual variation. Our approach offers a more effective avenue, allowing us to detect the original directions of postural instability resulting from dysfunction in the neuromuscular mechanisms that sustain an upright posture. Significantly, we demonstrated its ability to provide valuable insights into postural dyscontrol associated with Parkinson’s disease within 30 s of stabilography. The consistent outcomes across various contexts, laboratories, and clinics, irrespective of changes in visual input or postural difficulty, underscore our analysis’s high degree of reliability.

## Methods

Each patient gave informed written consent with full knowledge of the details. The ethics committee of the Federal University of ABC, Brazil, approved the research, which followed the guidelines stated in the Declaration of Helsinki. All data were fully anonymized before we accessed them.

### Participants

We applied the oriented fractal scaling component analysis (OFSCA) to the postural CoP trajectory of 32 Parkinson’s patients ($M \pm SD$ age: $66 \pm 10$ years; 8 women) in both on-medication and off-medication conditions, 22 healthy older adults ($67 \pm 8$ years; 11 women), and 27 healthy younger adults ($28 \pm 5$ years; 12 women) as they maintained a stable upright stance in different task conditions. Data for individuals with Parkinson’s disease were obtained from a publicly available gait dataset^77^ (https://doi.org/10.6084/m9.figshare.13530587), and data for healthy younger and older adults were obtained from another publicly available dataset^78^ (https://doi.org/10.6084/m9.figshare.4525082).

Before the experimental sessions, patients were required to maintain a stable dose of L-DOPA medication for at least one month. Two experimental sessions were conducted with individuals with Parkinson’s disease, one in the medication’s ON condition and the other in the medication’s OFF condition. Patients were required to take their dopaminergic medication 1 h before the session to ensure dosage stabilization and, in turn, to qualify as being in the ON condition. Conversely, during the OFF condition, participants were required to abstain from using any medication for Parkinson’s disease for at least 12 h. The order in which the “ON” and “OFF” sessions were conducted was randomized among the patients. The experimental sessions began at the same start time for all patients. These procedures were implemented to ensure that the data collected was reliable and accurately reflected the effects of L-DOPA medication on individuals with Parkinson’s disease.

### Experimental procedure

Balance in healthy adults and individuals with Parkinson’s disease was assessed by having them stand still for 30 s in four different conditions: standing on a rigid surface with eyes open, standing on a rigid surface with eyes closed, standing on an unstable surface with eyes open, and standing on an unstable surface with eyes closed. Each condition was repeated three times, and the order of the conditions was randomized for each individual. For the rigid surface conditions, under the foot, a $40\times 60$ cm force platform (OPT400600-1000; AMTI, Watertown, MA, USA). A 6-cm high foam block (Balance Pad; Airex AG, Sins, Switzerland) was placed on the force platform for unstable surface conditions. In all conditions, the individual stood barefoot, kept his/her arm at his/her sides, and looked at a 5 cm round black target on a wall 3 meters ahead at his/her eye level. For trials where the eyes were closed, instruction was given to first look at the target with his/her eyes open, find a stable and comfortable posture, and then close his/her eyes; the data acquisition began a few seconds later. During all trials, the individual’s feet were positioned at a 20-degree angle with their heels 10 cm apart by standing on lines marked on the force platforms and foam blocks. The trials were conducted in a room measuring $11.5\times 9.3$ m with white walls and sufficient lighting. The ground reaction force (GRF) data were collected at a sampling frequency of 100 Hz using the Cortex software version 7.0 (Motion Analysis, Santa Rosa, CA, USA).

### Data acquisition and processing

In the original studies^77,78^, Several steps were taken to process the data from the force platform. First, a fourth-order zero-lag low-pass Butterworth filter with a frequency of 10 Hz was applied to the force platform data to reduce noise. Next, CoP was calculated using the standard formula. The coordinate systems of the force platform were transformed into the laboratory coordinate system using transformation matrices in the Cortex software to integrate the CoP and GRF data. This transformation allowed for the data representation in the mediolateral, anteroposterior, and vertical components of the CoP, force, moment of force, and free moment of force (i.e., the moment around the normal to the force plate on the participant’s foot).

### Oriented fractal scaling component analysis (OFSCA)

So far we model CoP based on two assumptions: (1) that a 2D CoP planar trajectory can be fully characterized by considering two independent fBm sample paths, $\bigl \{(x^{(1)}[i]\bigl \}$ and $\bigl \{ x^{(2)}[i])\bigl \}$ ($i=1,2,\dots , N$; *N* is the length), and (2) that these two components are always orthogonal to each other. Thus, the scaling property of each angular component (projection onto a rotated direction) is identical and not affected by any rotational transform. This “isotropy,” however, might be an exception and not the rule, as no reason exists to believe that any natural trajectory should be isotropic^153,154^. We used the oriented fractal scaling component analysis^76^ to analyze the anisotropic autocorrelation properties of 2D CoP planar trajectories. This approach first evaluates the angle-dependent scaling properties of the trajectory using higher-order DMA^155^ (henceforth called DDMA). Then, it decomposes the observed 2D trajectory into two components with different orientations and scaling properties.

#### Angle-dependent scaling

The 2D CoP planar trajectory is projected onto a direction forming angle $\theta$ with the positive direction of the *x*-direction to detect the anisotropic scaling behavior. Projected time series $\{x^{(\theta )}[i]\}$ is given as

1 $$\begin{aligned} x^{\theta }[i]=x^{(1)}[i]\cos {\theta }+x^{(2)}[i]\sin {\theta }, \end{aligned}$$

where superscripts (1) and (2) represent the directions corresponding to the weakest and strongest postural control and $\theta$ is varied across the range $0\le \theta <\pi$ The projected time series $\bigl \{x^{(\theta )}[i]\bigl \}$ for each $\theta$ is subjected to the Savitzky–Golay-filter-based DMA^156,157^. The DDMA first integrates $\bigl \{x^{(\theta )}[i]\bigl \}$.

2 $$\begin{aligned} y^{\theta }[i]=\sum _{j=1}^{i}x^{\theta }[j]. \end{aligned}$$

The directional fluctuation function, $F^{(\theta )}(s)$, is then calculated as

3 $$\begin{aligned} F^{(\theta )}(s)=\sqrt{\frac{1}{N-s+1}\sum _{i=(s+1)/2}^{N-(s-1)/2} \Bigl (y^{(\theta )}[i]-\tilde{y}_{SG}^{(m,s)}[i]\Bigl )^{2}}, \end{aligned}$$

where $\tilde{y}_{SG}^{(m,s)}$ represents the locally smoothed version of $\bigl \{ y^{(\theta )}[i] \bigl \}$ obtained after applying the Savitzky–Golay filter with a polynomial of degree *m* and window length *s*. In Eq. (3), this locally smoothed version $\Bigl \{\tilde{y}_{SG}^{(m,s)}[i]\Bigl \}$ is removed from $\bigl \{y^{(\theta )}[i]\bigl \}$ to attenuate the baseline nonstationarity embedded in the obtained time series $\bigl \{y^{(\theta )}[i]\bigl \}$. Anisotropic autocorrelation properties can be evaluated using the $\theta$-dependent heterogeneity in the temporal correlation, $F^{(\theta )}(s)$, for each $\theta$ as

4 $$\begin{aligned} F^{(\theta )}(s)\sim s^{\alpha (\theta )}, \end{aligned}$$

quantified using the scaling exponent $\alpha (\theta )$ estimated as the slope of the double-logarithmic plot of $F^{(\theta )}(s)$ against *s*.

Note that $F^{(\theta )}(s)$ can be directly linked with auto-correlation function $C^{(\theta )}(k)$ and power spectrum $S^{(\theta )}(f)$ of $\bigl \{x^{(\theta )}[i]\bigl \}$^158,159^. That is,

5 $$\begin{aligned} F^{(\theta )}(s)=\sqrt{\sum _{k=-s}^{s}C^{(\theta )}(k)\,L(k,s)}=\sqrt{\int _{-1/2}^{1/2}\big |S^{(\theta )}(f)\big |\,|G_{s}(f)|^{2}df}. \end{aligned}$$

The analytical forms of kernels *L*(*k*, *s*) and $|G_{s}(f)|^{2}$ have been discussed in^158,159^. Using these relations in conjunction with Eq. (4), we can show that the scaling relations, $\alpha =1-\gamma /2$ when $C^{(\theta )}(k)\sim k^{-\gamma }(0<\gamma <1)$ and $\alpha =(\beta +1)/2$ when $S^{(\theta )}(f)\sim f^{-\beta }(-1<\beta <2m+3)$. In particular, when $C^{(\theta )}(k)$ decays exponentially to zero and $S^{(\theta )}(f)$ shows the low-frequency plateau indicating short-term correlation, the scaling exponent results asymptotically in $\alpha =0.5$.

Because the higher-order DMA induces timescale distortion between the scale in the time domain of DMA and the frequency in the Fourier spectral domain^159^, we used the corrected timescale, $\tilde{s}$, instead of *s*. Although scale *s* in the zeroth-order DMA corresponds well to frequency *f* in the Fourier spectral domain, i.e., $\tilde{s}=s/1.00$, $\tilde{s}$ is given by $\tilde{s}=s/1.93$ in the second order DMA. Because a straightforward implementation of the procedure has a high computational complexity, we employed a fast algorithm of DMA instead^155^.

#### Decomposition of mixed long-range correlated fluctuations

**Figure 7:**
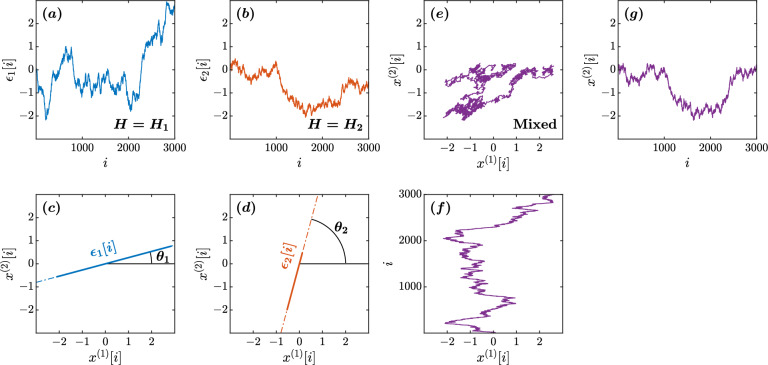
Illustration of a mixed fGn model with $H_{1}=0.60$, $\theta =\pi /12$, $H_{2}=0.40$, and $\theta _{2}=5\pi /12$. (a,b) The original components $\bigl \{\epsilon _{1}[i]\bigl \}$ and $\bigl \{\epsilon _{2}[i]\bigl \}$. (c,d) The orientations of $\bigl \{\epsilon _{1}[i]\bigl \}$ and $\bigl \{\epsilon _{2}[i]\bigl \}$ in the $\bigl (x^{(1)},x^{(2)}\bigl )$ plane. (e–g) Mixed fluctuations of $\bigl \{\epsilon _{1}[i]\bigl \}$ and $\bigl \{\epsilon _{2}[i]\bigl \}$ using Eq. (6) in the $\bigl (x^{(1)},x^{(2)}\bigl )$ plane.

Two independent fBm processes $\bigl \{\epsilon _{1}[i]\bigl \}$ and $\bigl \{\epsilon _{2}[i]\bigl \}$ with two different Hurst exponents $H_{1}$ and $H_{2}$ $(H_{1}>H_{2})$, respectively (Fig. 7a,b), oriented at angles $\theta _{1}$ and $\theta _{2}$, respectively (Fig. 7c,d), to the *x*-direction can be modeled to mix as

6 $$\begin{aligned} \begin{bmatrix} x^{(1)}[i] \\ x^{(2)}[i] \end{bmatrix} = \begin{bmatrix} \cos {\theta _{1}} &{} \cos {\theta _{2}} \\ \sin {\theta _{1}} &{} \sin {\theta _{2}} \end{bmatrix} \begin{bmatrix} \epsilon _{1}[i] \\ \epsilon _{2}[i] \end{bmatrix}, \end{aligned}$$

yielding the two independent fBm sample paths, $\bigl \{(x^{(1)}[i]\bigl \}$ and $\bigl \{x^{(2)}[i]\bigl \}$, respectively (Fig. 7e–g). In Eq. (6), the projected time series is given by

7 $$\begin{aligned} x^{(\theta )}[i]=\epsilon _{1}[i]\cos {(\theta -\theta _{1})}+\epsilon _{2}[i]\cos {(\theta -\theta _{2})}, \end{aligned}$$

indicating that, when $\theta =\theta _{1}+\pi /2$, $x^{(\theta )}[i]$ is orthogonal and independent of $\epsilon _{1}[i]$ (Fig. 1a). That is,

8 $$\begin{aligned} x^{(\theta _{1}+\pi /2)}[i]=\epsilon _{2}[i]\cos {(\theta _{1}-\theta _{2}+\pi /2)}, \end{aligned}$$

which is proportional to the original $\epsilon _{2}[i]$ with $H_{2}$. Likewise, $x^{(\theta _{2}+\pi /2)}[i]$ is orthogonal to $\epsilon _{2}[i]$ and proportional to the original $\epsilon _{1}[i]$ with $H_{1}$. That is,

9 $$\begin{aligned} x^{(\theta _{2}+\pi /2)}[i]=\epsilon _{1}[i]\cos {(\theta _{2}-\theta _{1}+\pi /2)}, \end{aligned}$$

In contrast, when $\theta \ne \theta _{1}\pm \pi /2$ and $\theta \ne \theta _{2}\pm \pi /2$,$F^{(\theta )}(s)$ shows a crossover of scaling exponents (Fig. 1b). The forced linear fit to the broken lines in the log-log plot of $F^{(\theta )}(s)$ vs. *s* yields a slope in the range $(H_{2}, H_{1})$. Therefore, seeking two main orientations, $\hat{\theta }_{min}$ and $\hat{\theta }_{max}$, respectively, with the minimum and maximum values of $\alpha (\theta )$, the original orientations of $\epsilon _{1}[i]$ and $\epsilon _{2}[i]$ can be estimated as $\hat{\theta }_{1}=\hat{\theta }_{min}\pm \pi /2$ and $\hat{\theta }_{2}=\hat{\theta }_{max}\pm \pi /2$, respectively (Fig. 1c). Thus, using the angles $\hat{\theta }_{2}$ and $\hat{\theta }_{2}$, the original 2D CoP planar trajectory can be estimated from the observed 2D CoP planar trajectory as

10 $$\begin{aligned} \begin{bmatrix} \hat{\epsilon }_{1}[i] \\ \hat{\epsilon }_{1}[i] \end{bmatrix} = \begin{bmatrix} \sin {(\hat{\theta }_{2})}/\sin {(\hat{\theta }_{2}-\hat{\theta }_{1})} &{} \sin {(\hat{\theta }_{2})}/\cos {(\hat{\theta }_{1}-\hat{\theta }_{2})} \\ \sin {(\hat{\theta }_{1})}/\sin {(\hat{\theta }_{1}-\hat{\theta }_{2})} &{} \sin {(\hat{\theta }_{1})}/\cos {(\hat{\theta }_{2}-\hat{\theta }_{1})} \end{bmatrix} \begin{bmatrix} x^{(1)}[i] \\ x^{(2)}[i] \end{bmatrix}. \end{aligned}$$

**Figure 8:**
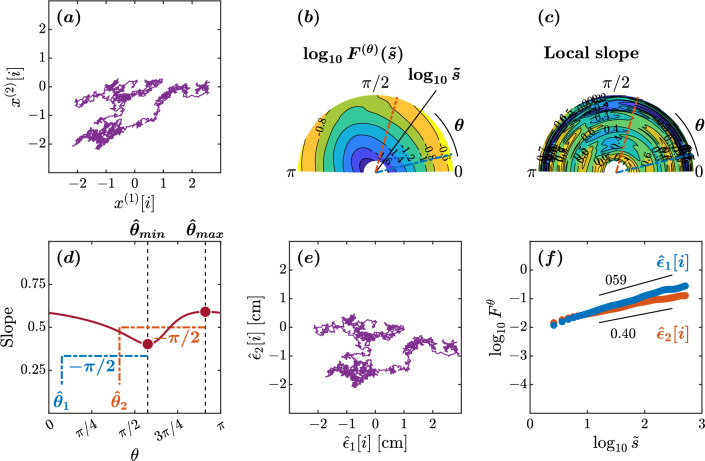
Orientation decomposition of the fBm trajectory shown in Fig. 7. (**a**) The two components $x^{(1)}[i]$ and $x^{(2)}[i]$ of the mixed fBm trajectory. (**b**) $\theta$-dependent heterogeneity in $F^{(\theta )}(s)$, indicated by the angle dependence of $\log _{10}^{(\theta )}F^{(\theta )}(\tilde{s})$ vs. $\log _{10}\tilde{s}$, where $\tilde{s}\sim s/1.93$ in the second order DDMA. (**c**) $\theta$-dependence of the local slopes of $\log _{10}^{(\theta )}F^{(\theta )}(\tilde{s})$ vs. $\log _{10}\tilde{s}$, indicating the spatial distribution of temporal correlations. (**d**) $\theta$-dependence of the slope in the range of $1.2<\log _{10}{\tilde{s}}<2.5$. (**e**) Reconstructed original components $\hat{\epsilon }_{1}[i],\hat{\epsilon }_{2}[i]$. (**f**) Fluctuation functions of the reconstructed original components $\hat{\epsilon }_{1}$ with $\hat{\theta }_{1}=14^{\circ }$ and $\hat{\epsilon }_{2}$ with $\hat{\theta }_{2}=74^{\circ }$.

To explain the OFSCA, we analyzed the sample fBm time series with $H_{1}=0.60$, $\theta _{1}=\pi /12$, $H_{2}=0.40$, and $\theta _{2}=5\pi /12$ (Figs. 7e,  8a) as a numerical illustration of the OFSCA. Figure 8b plots the angle dependence of $F^{(\theta )}(\tilde{s})$ over the range of $0\le \theta <\pi$ in increments of $\pi /179$ rad, in a circular cylindrical coordinate $(\rho ,\psi ,z)=(\log _{10}(\tilde{s}),\theta ,\log _{10}{F^{(\theta )}(\tilde{s}))}$, where $\rho$ is the distance of a coordinate point from the Cartesian *z*-direction, and $\psi$ is its azimuthal angle. In addition, Fig. 8c plots the local slopes of $\log _{10}{F^{(\theta )}(\tilde{s})}$ vs. $\log _{10}{\tilde{s}}$ at each $\theta$.

The apparent anisotropic scaling properties of fBm Fig. 8b,c can be observed in the [hypothetical] major and minor axes of the ellipse-shape variability in fBm Fig. 8a. Therefore, the conventional principal component analysis (PCA) cannot decompose the observed time series into the original orientations, as the variability cannot be decomposed into two orthogonal components. In contrast, the OFSCA allows the reconstruction of the original orientations of the 2D time series. In this approach, we first estimate $\theta _{min}=114^{\circ }$ and $\theta _{max}=164^{\circ }$ based on the angle dependence of the least-squares-fit slope as shown in Fig. 8d. Using Eq. (9) with $\theta _{1}=\theta _{min}-90^{\circ }=14^{\circ }$ and $\theta _{2}=\theta _{max}-90^{\circ }=74^{\circ }$, we decompose $\bigl \{\bigl (x^{(1)}[i],x^{(1)}[i]\bigl )\bigl \}$ into $\bigl \{\hat{\epsilon }_{1}[i]\bigl \}$ and $\bigl \{\hat{\epsilon }_{2}[i]\bigl \}$ (Fig. 8e). The estimated scaling exponents of $\bigl \{\hat{\epsilon }_{1}[i]\bigl \}$ and $\bigl \{\hat{\epsilon }_{2}[i]\bigl \}$ reproduced the theoretical values of 0.59 and 0.40 (Fig. 8f).

### Statistical analysis

We subjected each postural 2D CoP planar trajectory to the OFSCA and computed the angle between the major and minor axes of postural control as $\Delta \theta =\hat{\theta }_{1}\sim \hat{\theta }_{2}$. A linear mixed-effects model estimated changes in the dependent measure $\Delta \theta$ as a function of the following fixed effects: *Group* (a class variable defined with baseline value “YA” encoding healthy young adults and comparison of levels “OA,” “PN,” and “PM” encoding groups of healthy older adults, individuals with Parkinson’s disease “OFF” medication condition, and individuals with Parkinson’s disease “ON” medication condition, respectively), *Unstable* (equaling 0 or 1 when participants stood on the stable floor of the force platform or unstable foam surface, respectively), and *EyesClosed* (equaling 0 or 1 when participants stood with eyes open or eyes closed), as well as covariates $H_{1}$ and $H_{2}$ encoding the maximum and minimum strengths of temporal correlations on the corresponding trial. The model included additional fixed effects of the interactions of *Group* with *Unstable* and *EyesClosed*. To respect that our sample included a sex bias in the Parkinson’s disease group, alternate modeling also incorporated the covariate *Sex* (equaling 0 or 1 for women and men, respectively) both as a main effect and as an interaction with all other covariates. No main effect of *Sex* or interaction significantly improved model fit from the model reported in the present study, so we omitted *Sex* and its interactions from the model reported in the present report. We included the random factor of participant identity by allowing the intercept to vary across participants. Statistical analyses were performed in R^160^ using the function lmer from the package lme4^161^. Significance was set at the two-tailed $\alpha$ level of 0.05.

## Data Availability

Data for individuals with Parkinson’s disease were obtained from a publicly available gait dataset^77^ (https://doi.org/10.6084/m9.figshare.13530587), and data for healthy younger and older adults were obtained from another publicly available dataset^78^ (https://doi.org/10.6084/m9.figshare.4525082).
